# Gangrenous Erysipelas

**Published:** 1876-05

**Authors:** M. A. McClelland

**Affiliations:** Knoxville, Ill.


### GANGRENOUS ERYSIPELAS.

By M. A. McCLELLAND, M.D., Knoxville, Ill.

(Read before the Military Tract Medical Society, Jan. 11, 1876, at Galesburg, Ill.)

Case 1. Mrs. Hamerstrom, aged 50 years, while digging potatoes, wounded her foot very slightly with the tine of a potatoe fork. Three days after I was called in to see her, and found the foot swollen, hot, red and tender. At a point near the ankle, and at another near the seat of wound, there were appearances that led me to suppose one or two abscesses were forming. I the more readily made out this diagnosis, as the tissues were very tense, and did not pit under pressure.

Directed a saline cathartic, and applied locally lead water and laudanum.

The next morning the redness was more intense, and had extended up the limb two-thirds of the way to the knee; foot pitting.

Directed, the salts having acted well, tincture iron and quinine, gtt. xx, gr. ij, every three hours. Limb to be painted with tincture iodine; lead water and laudanum continued; morphine. Third day, a large blister had formed upon foot and ankle. Stopped local applications of iodine, lead water, etc., and directed a solution of pot. permanganate ʒij, ad aq. ℥j, with which foot was to be constantly wet.

The discharge from the wound, and also the contents of the blister, were a thin, watery fluid, containing flocculent, grayish matter. Morphine continued, to procure sleep and allay pain, which, however, at no time was very severe.

4th day. Foot and limb, as high as the knee, blistered. The surface around ankle, which first blistered, is becoming gangrenous. Quinine and iron continued. Limb to be kept constantly wet with solution of pot. permang.

5th day. Gangrene extending. A dense, gray slough forming at bottom of blisters. Treatment continued, with addition of egg-nog.

7th day. Gangrene does not spread. It now includes all the external half of the dorsum of foot from around the ankle to the toes. Several smaller patches upon the inside of the dorsum. Deep, grayish sloughs have cut off small islands of semi-healthy tissue, and are undermining them. Several have been thus separated from below, and lie upon the sloughs as black, fleshy masses. Saturated solution of bromine applied every hour. Iron and quinine continued, also stimulants and concentrated nourishment.

9th day. Sloughs separating. Red granulations appearing at edges of ulcers. Treatment continued.

12th day. Sloughs being thrown off in large masses. Dorsalis pedis lies uncovered for the space of an inch, and is covered with healthy granulations. Locally a weaker solution of bromine, the parts to be first cleansed with pot. permang. ʒj, aq. ℥j.

The above patient was an enfeebled, broken-down woman. Her hygienic surroundings were pretty good. The case was marked throughout by a very regular circulation, and with but slight febrile manifestations. Pulse never went to a hundred. Tongue remained moist throughout. No delirium. Prognosis now is quite favorable.

CASE 2. S. M., aged 52 years. Was called to see patient July 31, 1875. He gave the following commemorative history: Was pitching hay during the week, and one day, while it was raining, he went to sleep in the hay mow, and slept most of the afternoon. Woke up with headache and a good deal of pain about the left pectoral muscle. Headache and pain still continued, in addition to which he was now feverish; anorexia marked; no chills. Had taken some pills, which had operated.

I found him with a dry, red tongue, pulse 90 per minute, somewhat feverish, and with considerable soreness in left pectoral muscle. Diagnosed a threatened attack of typho-malarial fever, with perhaps some rheumatic trouble about pectoralis.

Left him bromide of potass. for headache, and quinine gr. v. every three hours. He was to have his clothing and bed-linen changed every day, and to be well sponged off with water of such a temperature as was most agreeable several times a day.

I saw him again the next day, and found no change. Treatment continued, with the addition of acid. sulph. aromat., ten drops with each quinine powder.

Saw him again August 3d, when I found him free from headache. Soreness had left breast and had settled in the left lumbar region, which, upon examination, was found to be swollen. He was directed to poultice the part and continue the quinine and acid in smaller doses. Was not to see him again till the 5th, but on the morning of the 4th he sent for me on account of retention of his urine. He had taken gr. ¼ of morphine the night before, for the purpose of promoting sleep. I introduced a No. 10 catheter and drew off a quart of water, which presented a healthy appearance, except it was slightly darker than normal. Treatment continued, with the addition of a mild cathartic. Lumbar abscess diagnosed.

On the 5th of August cathartic had acted. Appearance of an abscess more marked.

Dr. Reece, of Abington, Ill., was sent for and saw him the morning of the 6th, and confirmed the diagnosis. Advised iron to be added to quinine. Patient's urine had still to be drawn with catheter. The sense of fluctuation was very obscure, and inasmuch as patient was not suffering much, he was advised to continue poultice another day. He had taken two pills the night before, at his own suggestion, and had reported them this morning. These had not yet operated.

On the 9th day (Aug. 7), there appeared upon his back, at some distance from the focus of swelling, two black spots about half an inch in diameter. Over the swelling the skin had become brawny, and a number of yellowish pimples appeared, just as in cases of carbuncle. He was ordered to continue treatment, and to take an abundance of nourishment and egg-nog. Pulse about 100 per minute.

10th day. The black spots had extended so as to include the entire lumbar region on both sides. But little pain. Retention of urine continued. Carbolic acid ointment (very weak) was spread upon a cloth and applied to the back, to prevent his clothes from sticking.

11th day. A space ten by twelve inches was denuded of cuticle; a slough about four by five inches was forming in the true skin on the right side; pulse 145; extremities cold; cold, clammy sweat. Diarrhœa set in during the night. Died at 2 o'clock P. M.

This patient was somewhat worn down by hard work and dyspepsia. His case, from the start, was marked by very slight delirium up to the day he died, when it became a decided feature. Both the cases were singular, in the fact that but little pain was complained of.

At the present writing, from notes made at the time, Case 1 has fully recovered, and before closing the paper will add another case, which is yet under observation.

CASE 3.—S. S., farmer, age about 65, consults me in regard to his thumb, which has been swollen some two or three weeks, and supposing it to be of the nature of a felon, has been poulticing it, and a day or two previous has had it partly opened.

Being very busy, I kept no notes of the case, nor record of visits, although these, for the greater part of three weeks, were made twice and three times a day.

Upon my first visit, the appearances were so much those of a phlegmonous inflammation I carried a scalpel down to the first phalanx of the thumb, making an incision half or three-quarters of an inch in length. Only a little unhealthy pus was evacuated, and this apparently from just beneath the true skin. This incision, during the next three or four days, was carried through the true skin, across to and down the index finger to the junction of the second and third phalanx. The inflammation extended to the second and third fingers, the palm of the hand, and up the arm. The patient being feeble, he took constantly iron and quinine, and had advised for him egg-nog, but took none of this till the second week of my attendance. Incisions were made in various parts of the hand and fingers, being carried down to the bone in places, but the entire suppurative action seemed to be located in the subcutaneous connective tissue and skin. During the first week, gangrene set in, in the end of the thumb and index finger. Solutions of carbolic acid, bromine and permanganate of potash, were constantly used. To the washing out of the deeper diseased parts I attended myself. The connective tissue in the arm suppurated to within three or four inches of the elbow, mainly upon the palmar surface of the arm. Lengthy incisions gave exit to no more pus than shorter ones, through the local pockets. For the last few weeks of the suppurative action, only short incisions were practiced. During the second or third week, the entire fore-finger seemed to be gangrenous, as well as the end of the thumb. Intense pain required the constant use of morphine. About the fourth week the inflammation began to subside in the arm, and finally subsided, to some extent, in the hand and fingers. The sixth week I amputated the fore-finger through the second phalanx, since which time there has been a steady but very slow subsidence of the inflammation. Some of the gangrenous spots have not yet healed, and are still having applied to them the solution of bromine, but the patient is convalescing, and will shortly be well.

